# Attribute Index and Uniform Design Based Multiobjective Association Rule Mining with Evolutionary Algorithm

**DOI:** 10.1155/2013/259347

**Published:** 2013-04-28

**Authors:** Jie Zhang, Yuping Wang, Junhong Feng

**Affiliations:** ^1^School of Computer Science and Technology, Xidian University, Xi'an 710071, China; ^2^Department of Computer Science and Technology, Guangzhou University Sontan College, Zengcheng, Guangzhou 511370, China

## Abstract

In association rule mining, evaluating an association rule needs to repeatedly scan database to compare the whole database with the antecedent, consequent of a rule and the whole rule. In order to decrease the number of comparisons and time consuming, we present an attribute index strategy. It only needs to scan database once to create the attribute index of each attribute. Then all metrics values to evaluate an association rule do not need to scan database any further, but acquire data only by means of the attribute indices. The paper visualizes association rule mining as a multiobjective problem rather than a single objective one. In order to make the acquired solutions scatter uniformly toward the Pareto frontier in the objective space, elitism policy and uniform design are introduced. The paper presents the algorithm of attribute index and uniform design based multiobjective association rule mining with evolutionary algorithm, abbreviated as IUARMMEA. It does not require the user-specified minimum support and minimum confidence anymore, but uses a simple attribute index. It uses a well-designed real encoding so as to extend its application scope. Experiments performed on several databases demonstrate that the proposed algorithm has excellent performance, and it can significantly reduce the number of comparisons and time consumption.

## 1. Introduction

Data mining is a very active and rapidly growing research area in the field of computer science. Its aim is to extract interesting and useful knowledge from a huge number of data stored in the databases. Association rule mining is one of the most well-known data mining technologies. It can find out effectively interesting relations among attributes.

Existing algorithms for mining association rules are mainly based on the approach suggested by Agrawal and Srikant [1, 2]. Apriori [2], SETM [3], AIS [2], Pincer search [4, 5], DIC [6], and so forth are some of the popular algorithms based on this approach. 

The above algorithms can find out massive amount of possible rules. However, a large number of rules will increase the complexity and make the rule set harder to understand by users. That is to say, the greater the number of rules in the results is, the greater the complexity for the users is [7]. Therefore, generating the rules which are as valid and few as possible is our ultimate aim. How to select representative and useful rules and to remove those similar rules is our greatest concern. In order to deal with the above problems, this paper introduces elitism policy and uniform design. 

In the meanwhile, the above algorithms depend on two user-predefined parameters, *minimum support* and *minimum confidence*. However, how to select them is not an easy issue. If the value of *minimum support* is too large, the number of frequent itemsets generated will be less, and thereby too few rules may be generated. By contrast, if the value is too small, then almost all possible itemsets will become frequent and thus a huge number of rules may be generated. Similarly, if the value of *minimum confidence* is too large, many generated rules will be removed, and thereby some useful rules may be missing. However, if the value is too small, then almost all possible rules will become *strong rules* and thus a huge number of rules may be generated. Therefore, multiobjective rule mining with evolutionary algorithm is introduced, which visualizes rule mining problem as a multiobjective problem rather than a single objective problem. It need not specify those two user-predefined parameters any further [8–10]. 

Association rule mining algorithms can be taken into two steps. First they find the frequent itemsets and then extract the important association rules from the frequent itemsets. Among the two steps, the first step is the most time-consuming [7]. The reason is that in order to evaluate an association rule of the form *X* → *Y*, we need to repeatedly scan the database to compare to the whole database with *X*, *Y*, and *X* ∪ *Y* itemsets [11]. In this paper, we present an attribute index method to decrease the number of comparisons. It is remarkable that the proposed method scans database only once. 

The rest of this paper is organized as follows.  Section 2 states the preliminaries of the proposed method.  Section 3 presents our method in detail.  Section 4 gives the numerical results of the proposed method. The conclusion of the work is made in Section 5.

## 2. Preliminaries

In this section, we describe some concepts concerning association rule, multiobjective evolutionary algorithms, uniform design, and multiobjective association rule mining.

### 2.1. Association Rules and Metrics

Let *I* = {*i*_1_, *i*_2_, *i*_3_,…, *i*_*m*_} be a set of items or itemset. Let *D* = {*T*_1_, *T*_2_,…, *T*_*n*_} be the set of transactions, called the *transaction database*, where each transaction *T*_*i*_ ∈ *D* is an itemset such that *T*_*i*_⊆*I*. An association rule is of the form *X* → *Y* where *X*⊆*I*, *Y*⊆*I*, and *X*∩*Y* = Φ. The itemsets *X*, *Y* are respectively called the antecedent and consequent of the association rule.

A transaction *T*_*i*_ contains an itemset *X*, *T*_*i*_⊇*X*, if and only if, for any item *i* ∈ *X*, then *i* ∈ *T*_*i*_, namely, *T*_*i*_, contains each item in *X*.

*Support count * of an itemset *I*_1_ is denoted by SUP(*I*_1_), which is the number of transactions that contain *I*_1_ in *D*:

(1) $$\begin{array}{c} \text{SUP}\left(I_{1}\right)=\left|\left\{t\in D\wedge t⊇I_{1}\right\}\right|. \end{array}$$

*Support count* of an association rule *X* → *Y* is denoted by SUP(*X* → *Y*), which is the number of transactions compatible with both *X* and *Y*, namely, the number of transactions that contain *X* ∪ *Y*:

(2) $$\begin{array}{c} \text{SUP}\left(X\to Y\right)=\text{SUP}\left(X\cup Y\right). \end{array}$$

In a similar way, SUP(*X*) and SUP(*Y*) are the number of transactions compatible with only *X* and *Y*, respectively. 

*Support* of an itemset *I*_1_ is denoted by support (*I*_1_), which is the ratio of transactions that contain *I*_1_ in *D*, namely,

(3) $$\begin{array}{c} \text{support}\left(I_{1}\right)=\frac{\text{SUP}\left(I_{1}\right)}{\left|D\right|}, \end{array}$$

where |*D*| indicates the total number of transactions in the database *D*.

*Support* of an association rule *X* → *Y* is denoted by support(*X* → *Y*):

(4) $$\begin{array}{c} \text{support}\left(X\to Y\right)=\frac{\text{SUP}\left(X\to Y\right)}{\left|D\right|}=\frac{\text{SUP}\left(X\cup Y\right)}{\left|D\right|}. \end{array}$$

An itemset, *X*, in a transaction database, *D*, is called a large (frequent) itemset if its Support is larger than or equal to a threshold of minimum support (minsupp), which is given by users or experts.

The *confidence* or *predictive accuracy* of a rule *X* → *Y*, written as confidence(*X* → *Y*), is to measure specificity or consistency. It indicates the probability of creating the rule dependent on the antecedent part, and is defined as follows:

(5) confidence(X→Y)  =support(X→Y)support(X)=SUP(X∪Y)SUP(X).

That is, *support* implies frequency of cooccurring patterns, and *confidence* means the strength of a rule. The *support-confidence framework *is as follows [1, 2].

The minimal support, minsupp, and the minimal confidence, minconf, are given by users or experts. Then rule *X* → *Y* is valid if

(6) $$\begin{array}{c} \text{support}\left(X\to Y\right)\geq \text{minsupp},\\ \text{confidence}\left(X\to Y\right)\geq \text{minconf}. \end{array}$$

Generally speaking, only those rules with *support* and *confidence* larger than or equal to a given threshold are interesting. These rules are called *strong rules*.

Mining association rules can be taken into the following two subproblems.

1. Generating all itemsets whose *support* are greater than or equal to the user-specified minimum support, that is, generating all frequent itemsets.
2. Generating all the rules which satisfy the minimum confidence constraint. If the confidence of a rule is greater than or equal to the minimum confidence, then the rule can be extracted as a valid rule [8–10].

Apart from the above metrics, other several important metrics are illustrated as follows.

*Coverage* of an association rule *X* → *Y*, denoted by coverage(*X* → *Y*), is to measure the extent to which the consequent part is covered by the rule (the maximum value is reached when all the elements that satisfy *Y* are covered by the rule) [7]. It shows the probability of creating the rule dependent on the consequent part, and is defined as follows:

(7) $$\begin{array}{c} \text{coverage}\left(X\to Y\right)=\frac{\text{support}\left(X\to Y\right)}{\text{support}\left(Y\right)}=\frac{\text{SUP}\left(X\cup Y\right)}{\text{SUP}\left(Y\right)}. \end{array}$$

Both the *confidence* and *coverage* are two important measuring factors for the rule quality or rule interest, but if we use them separately we will reach bad conclusions [7]. 

The generated rules may have a large number of attributes involved, which may make them difficult to understand [12]. If the generated rules are not understandable to the users, the users will never use them. A careful study of an association rule infers that if the number of conditions involved in the antecedent part is less, the rule is more comprehensible. Therefore, *comprehensibility* of a rule *X* → *Y* can be measured by the number of attributes involved in the rule. It is quantified by the following expression [8, 9]:

(8) $$\begin{array}{c} \text{comprehensibility}=\frac{\log\left(1+\left|Y\right|\right)}{\log\left(1+\left|X\cup Y\right|\right)}. \end{array}$$

Here, |*Y*| and |*X* ∪ *Y*| are the number of attributes involved in the consequent part and the whole rule, respectively.

Another *comprehensibility* of a rule is defined as follows [13]:

(9) $$\begin{array}{c} \text{comprehensibility}=1-\frac{n}{N}, \end{array}$$

where *n* and *N* are, respectively, the numbers of attributes in antecedent part and in the whole dataset. 

*Comprehensibility* of a rule tries to increase the readability by shortening the length of an association rule.

*Interestingness* of a rule, denoted by interestingness(*X* → *Y*), is used to quantify how much the rule is surprising for the users. As the most important purpose of rule mining is to find some hidden information, it should extract those rules that have comparatively less occurrence in the database. The following expression can be used to quantify the interestingness [8, 9, 14, 15]:

(10) Interestingness(X→Y)  =SUP(X∪Y)SUP(X)×SUP(X∪Y)SUP(Y)   ×(1−SUP(X∪Y)|D|),

where |*D*| indicates the total number of transactions in the database. 

Yan et al. defined the relative confidence as the interestingness measure as follows [10]:

(11) $$\begin{array}{c} \text{rconf}=\frac{\mathrm{supp}\left(X\cup Y\right)-\mathrm{supp}\left(X\right)\mathrm{supp}\left(Y\right)}{\mathrm{supp}\left(X\right)\left(1-\mathrm{supp}\left(Y\right)\right)}. \end{array}$$

Here, supp indicates *support*.

Hipp et al. [16] compared the popular association rule mining approaches including Apriori [1, 2], Partition [17], and Eclat [18] and made conclusions that these approaches have shown similar runtime behavior. They found no algorithm that fundamentally outperforms others. For example, Apriori is superior in the market basket database, but it performs poorly with the car equipment database. The FP-growth algorithm is very efficient in many cases, but it requires a large amount of memory to store the FP-tree [19]. Although there may be differences with different implementations and datasets, association rule mining approaches have the same performance behavior with respect to the support threshold value. The experiments conducted in articles [1, 16, 20, 21] have shown that the decrease of the support threshold leads to an exponential increase on the number of frequent itemsets, which consequently results in an exponential increase in runtime and resource usage (i.e., memory and disk space) during the frequent itemset mining process [22].

### 2.2. Multiobjective Evolutionary Algorithms

The notion of Pareto-optimality is one of the major approaches to multiobjective programming. For any two points *x*_1_ and *x*_2_ in *Ω*, if the following conditions hold:

(12) $$\begin{array}{c} f_{i}\left(x_{1}\right)\leq f_{i}\left(x_{2}\right),\;\text{for}\;\;\text{all}\;\;i\in \left\{1,2,\ldots ,M\right\},\\ f_{j}\left(x_{1}\right)<f_{j}\left(x_{2}\right),\;\text{for}\;\text{some}\;\;j\in \left\{1,2,\ldots ,M\right\}, \end{array}$$

then *x*_1_ is at least as good as *x*_2_ with respect to all the objectives (the first condition), and *x*_1_ is strictly better than *x*_2_ with respect to at least one objective (the second condition). Therefore, *x*_1_ is strictly better than *x*_2_. If no other solution is strictly better than *x*_1_, then *x*_1_ is called a *Pareto-optimal solution*. A multiobjective programming problem may have multiple Pareto-optimal solutions, and these solutions can be regarded as the best compromise solutions. Different decision-makers may select different Pareto-optimal solutions in terms of the preference for themselves. It may be desirable to find all the Pareto-optimal solutions, so that the decision-makers can select the best one based on his preference. The set of all possible Pareto-optimal solutions constitutes a *Pareto frontier* in the objective space. 

Many multiobjective programming problems have very large or infinite numbers of Pareto-optimal solutions. When it is not possible to find all these solutions, it may be desirable to find as many solutions as possible in order to provide more choices to the decision maker [23].

Evolutionary algorithms, EAs, simultaneously deal with a set of possible solutions, which allows finding several members of the Pareto optimal set in a single run of the algorithm. Additionally, they are not too susceptible to the shape or continuity of the Pareto front (e.g., they can easily deal with concave and discontinuous Pareto fronts).

Schaffer is generally considered as the first to design a Multiobjective Evolutionary Algorithms (MOEAs), during the mid-1980s [24]. However, it was until the mid-1990s that MOEAs started to attract serious attention from researchers. Nowadays, it is possible to find applications of MOEAs in almost all domains [25].

Schaffer's approach, called Vector Evaluated Genetic Algorithm (VEGA), consists of a simple genetic algorithm with a modified selection mechanism. After VEGA, there has been a growing interest in applying evolutionary algorithms to deal with multiobjective optimization. The researchers designed a first generation MOEAs where the main lesson learned was that successful MOEAs had to combine a good mechanism to select non-dominated individuals. The most representative algorithms of this generation MOEAs are as follows: Non-dominated Sorting Genetic Algorithm (NSGA) [26], Niched-Pareto Genetic Algorithm (NPGA) [27], and Multi-Objective Genetic Algorithm (MOGA) [28]. A second generation MOEAs started when elitism became a standard mechanism. In fact, the use of elitism is a theoretical requirement in order to guarantee convergence of a MOEA. Many MOEAs have been proposed during the second generation. The Strength Pareto Evolutionary Algorithm  2 (SPEA2) [29] and the NSGA-II [30] can be considered as the most representative MOEAs of the second generation [31]. There are many works about MOEAs published every year. Zhou et al. surveys the development of MOEAs primarily during the last eight years [32]. The paper indicates that more than 5600 publications have been published on evolutionary multiobjective optimization By January 2011. Among these papers, 66.8% have been published in the last eight years, 38.4% are journal papers, and 42.2% are conference papers.

### 2.3. Uniform Design

The main objective of uniform design is to sample a small set of points from a given set of points, such that the sampled points are uniformly scattered [23, 33–35].

Let there be *n* factors and *q* levels per factor. When *n* and *q* are given, the uniform design selects *q* from *q* ^*n*^ possible combinations, such that these *q* combinations are uniformly scattered over the space of all possible combinaions. The selected *q* combinations are expressed as a uniform array *U*(*n*, *q*) = [*U*_*i*,*j*_]*q* × *n*, where *U*_*i*,*j*_ is the level of the *j*th factor in the *i*th combination, and can be calculated by the following formula:

(13) $$\begin{array}{c} U_{i,j}=\left(i\sigma^{j-1}\mathrm{mod}\;q\right)+1, \end{array}$$

where *σ* is a parameter given in Table 1. 

### 2.4. Multiobjective Association Rule Mining with Evolutionary Algorithm

The rules produced by the rule mining approach need to be evaluated using various metrics like the support and confidence. There are also other metrics such as the comprehensibility and interestingness of the rules. These make the rules more usable. If these metrics are consistent, they can be merged. However, the metrics are conflicting sometimes. For example, a user may wish to have rules which are both novel and accurate. However, these two objectives are conflicting since if the accuracy of the rule increases its novelty will decrease. Thus the problem of constructing rules with specific metrics should be faced as a multiobjective optimization problem [36].

In the early years, some optimization methods for association rule mining have been proposed. However, the process is too much resource consuming, especially when there is not enough available physical memory for the whole database. A solution to this problem is to use evolutionary algorithm, which reduces both the cost and time of rule discovery. Evolutionary algorithm (EA), genetic algorithm (GA), ant colony optimization (ACO), and particle swarm optimization (PSO) are instances of single objective association rule mining algorithms. A few of these algorithms have been used for multiobjective problems [9]. 

Multiobjective association rule mining with EA is to use EA to solve the association rule mining problem. Those metrics mentioned in Section 2.1 can be taken as multiply objectives to optimize in multiobjective rule mining. The operators such as select, crossover, and mutate are used to evolve the chromosome representing an association rule.

### 2.5. Related Works

There have been some attempts and works for multiobjective association rule mining using evolutionary algorithms. Ghosh and Nath visualized an association rule mining as a multiobjective problem rather than a single objective one [8], where multiobjective genetic algorithm, MOGA, was applied to maximize the confidence, comprehensibility and interestingness of a rule. Khabzaoui et al. used a parallel MOGA to optimize the support, confidence, *J*-measure, interest, and surprise [37]. Dehuri et al. presented an elitist MOGA for mining classification rules, which take three conflicting metrics with each other, accuracy, comprehensibility, and interestingness, as multiply objectives [38]. Iglesia et al. used multiobjective evolutionary algorithm to search for Pareto-optimal classification rules with respect to support and confidence for partial classification [39]. A multiobjective evolutionary algorithm combined with improved niched Pareto genetic algorithm was applied to optimize two conflicting metrics with each other, predictive accuracy and comprehensibility of the rules in multiobjective rule mining [40]. Rule mining method with PSO, chaos rough particle swarm algorithm [41], and numeric rule mining method with simulated annealing [42] have been proposed. Alatas et al. proposed multiobjective differential evolution algorithm for mining numeric association rules [43]. Later, they proposed another numeric association rule mining method using rough particle swarm algorithm. Yan et al. proposed a method based on genetic algorithm without considering minimum support [10]. Qodmanan et al. applied MOGA to association rule mining without taking the minimum support and confidence into account by applying the FP-tree algorithm [9]. Hoque et al. presented a method to generate both frequent and rare itemsets using multiobjective genetic algorithm [14]. Fung et al. suggested a novel MOGA based rule mining method for affective product design, which can discover a set of rules relating design attributes with customer evaluation based on survey data [44].

## 3. The Proposed Method

Section 2.1 has described several import metrics for the evaluation of an association rule. As using separately the *confidence* and *coverage* of a rule can reach bad conclusions [7], the two metrics are selected together in the proposed method. If the generated rules are not understandable to the users, they will never use them. Therefore, the *comprehensibility* of a rule is selected. As the most important purpose of association rule mining is to find some hidden information, therefore the *interestingness* of a rule is selected to quantify how much the rule is surprising for the users. The proposed method selects the four metrics as multiple objectives to optimize. Namely, we need to optimize the following multiobjective problem:

(14) Maximize confidenceMaximize coverageMaximize comprehensibilityMaximize interestingness.

### 3.1. Attribute Index

In the above four metrics, 3 of them need to calculate the *support count* of a rule. The *support count* of an itemset *X* is the number of transactions that contain *X* in *D*. A transaction *T*_*i*_ contains an itemset *X*, if and only if, for any item *i* ∈ *X*, then *i* ∈ *T*_*i*_, namely, *T*_*i*_, contain all the items in *X*. Therefore, to evaluate an association rule *X* → *Y*, the database *D* will be repeatedly scanned to compare each transaction *T*_*i*_ ∈ *D* with an itemset *X*, *Y* and *X* ∪ *Y*. In order to judge whether a transaction *T*_*i*_ contains an itemset *X* or not, we need to judge whether *T*_*i*_ contains each of the items of itemset *X*. Namely, the number of comparisons for an itemset *X* is formulated as follows:

(15) $$\begin{array}{c} {\text{NC}}_{X}=\left|D\right|\times \left|X\right|, \end{array}$$

where NC_*X*_ indicates the number of comparisons for an itemset *X*, |*D*| indicates the total number of transactions in the database *D*, and |*X*| indicates the number of all items in the itemset *X*. 

Therefore, the number of comparisons for a rule *X* → *Y* is formulated as follows:

(16) NCX→Y=NCX+NCY+NCX∪Y=|D|×(|X|+|Y|+|X∪Y|).

In the above formula, |*X*|, |*Y*|, and |*X* ∪ *Y*| indicate the number of the items in the antecedent, consequent, and the whole rule, respectively. If any of them turns less, the number of comparisons for a rule will turn smaller. In the meanwhile, from (8) and (9), we can see that the *comprehensibility* of a rule will also turn smaller. Namely, the smaller the size of the itemsets in a rule is, the more easily comprehensible the rule is and the smaller the number of comparisons is. In other word, selecting the more easily comprehensible rule can decrease the number of comparisons.

As |*D*| is fixed, we cannot decrease the number of comparisons through the parameter. However, if only by means of comparing a part of transactions rather than all transactions in *D*, we can still evaluate an association rule and calculate metrics values, then the number of comparisons can certainly decrease. 

Hadian et al. presented a method that only compares the transactions the size of which is larger than or equal to the size of the itemset, which is in terms of the fact that a transaction contains an itemset only if the minimal size of the transaction is equal to size of the itemset [11]. This method can prevent some unnecessary comparisons by excluding the transactions whose size is less than the size of the itemset. However, a majority of transactions that do not contain the itemset are still compared. An example is illustrated as follows.

**Example 1**

Assume a transaction database *D* contain 6 transactions, as shown in Table 2. The universal itemset *I* contains 6 attributes {*i*_1_, *i*_2_, *i*_3_, *i*_4_, *i*_5_, *i*_6_}. For a rule of the form *i*_1_ *i*_3_ *i*_5_ → *i*_4_, the above method can exclude *T*_1_ and *T*_2_ as their sizes are less than the size of the rule. It compares *T*_3_, *T*_4_, *T*_5_ and *T*_6_ with the rule. However, it is obvious that *T*_3_, *T*_4_, *T*_5_, are unsuitable as they miss a certain item of the rule, are impossible to contain the rule.

In order to overcome the above problems, the work presents the strategy of the attribute index. It can prevent a great deal of unnecessary comparisons by only comparing those transactions directly related to the rule. Therefore, it can significantly improve the performance of an algorithm.

The strategy creates the attribute index for each attribute in database. Its index value is the successive link of all transactions containing the attribute. For example, *T*_1_, *T*_2_, *T*_3_, *T*_4_, *T*_5_, and *T*_6_ can be previously defined as 1, 2, 3, 4, 5, 6 or 0, 1, 2, 3, 4, 5, and so on. The attribute index of the above example is as follows. The attribute index of the attribute *i* can be formulated as follows:

(17) $$\begin{array}{c} \text{Idx}\left(i\right)=\left\{k\;|\;i\in T\wedge T\in D\wedge T\;\text{is defined as}\;k\right\}. \end{array}$$

**Example 2**

For Table 2, the attribute index of each attribute is as follows:

(18) Idx(i1)={1,3,5,6};  Idx(i2)={3,4};Idx(i3)={1,4,5,6};Idx(i4)  ={2,3,4,6};  Idx(i5)={1,2,3,4,5,6};Idx(i6)={5,6}.

In this method, the database is scanned once to create the attribute index of each attribute before rule generations. The pseudocode of creating the attribute index is shown in Pseudocode 1. 

The created attribute indices make it easy to calculate the *support count* of the antecedent, consequent, and the whole rule. Therefore, several import metrics to evaluate a rule can also be easy to calculate as these calculations do not need scan a database anymore. The calculations of the *support count* of an itemset only acquire the same values of the attribute index of each item in the itemset. As the same values represent those transactions that contain the itemset, therefore, the number of the same values is just the *support count* of the itemset. The pseudocode of calculating the *support count* of an itemset is shown in the function SUPItem of Pseudocode 2. 

To calculate the *support count* of an association rule *X* → *Y*, we can take *X*, *Y* and *X* ∪ *Y* as an itemset or parameter to call the function SUPItem so as to calculate the *support count* of the antecedent, consequent, and the whole rule. The pseudocode of calculating the *support count* of an association rule is shown in the function SUPRule of Pseudocode 2.

**Example 3**

For a rule of the form *i*_1_ *i*_3_ *i*_5_ → *i*_4_, the attribute indices of each item *i*_1_, *i*_3_, and *i*_5_ in the antecedent part are Idx(*i*_1_) = {1,3, 5,6}; Idx(*i*_3_) = {1,4, 5,6}; Idx(*i*_5_) = {1,2, 3,4, 5,6}. The same values of them are {1,5, 6}. This indicates *T*_1_, *T*_5_, and *T*_6_ contain the antecedent of the rule, as can be verified from Table 2. Therefore, SUP(*i*_1_ *i*_3_ *i*_5_) = |{1,5, 6}| = 3. In the similar way, SUP(*i*_4_) = |{2,3, 4,6}| = 4; SUP(*i*_1_ *i*_3_ *i*_5_ → *i*_4_) = SUP(*i*_1_ *i*_3_ *i*_5_ ∪ *i*_4_) = |{1,5, 6}∪{2,3, 4,6}| = |{6}| = 1.

If the *support count* of the antecedent, consequent, and the whole rule is known, the *confidence* and *coverage* of the rule can easily be acquired according to (5) and (7). Formula (8) or (9) can easily calculate the *comprehensibility* of the rule in terms of the number of attributes involving in the consequent part and the whole rule.

The *interestingness* of an association rule can be calculated by (10) or (11). However, we can obviously see that the acquired *interestingness* according to (11) may be less than 0 because it is possible that supp⁡(*X* ∪ *Y*) is less than supp⁡(*X*)supp⁡(*Y*). The negative *interestingness* does not meet our requirements. Therefore, (11) is not what we need. For (10), we can deduce it as follows:

(19) interestingness(X→Y) =SUP(X∪Y)SUP(X)×SUP(X∪Y)SUP(Y)×(1−SUP(X∪Y)|D|) =confidence×coverage×(1−support).

From (19), we can obviously see that the *interestingness* of an association rule consists of 3 parts, the *confidence*, *coverage,* and complement of the *support*. Among them, the confidence and coverage are both larger than 0 and less than or equal to 1, and their product is also larger than 0 and less than or equal to 1. However, if they are very small, their product will be a great deal less than any of them. For instance, confidence = 0.3, coverage = 0.5, their product 0.15 is much less than 0.3 and 0.5. Therefore, the interestingness of a rule is often rather small. This has been confirmed by the results of many works. 

According to the definition of the interestingness of a rule, it is to extract the rules that have comparatively less occurrence in the database. Namely, the interestingness is to mine such association rules as low support but higher confidence. Therefore, we revise (19) as follows:

(20) interestingness(X→Y)  =α×confidence+β×confidence×(1−support)  =α×SUP(X∪Y)SUP(X)+β×SUP(X∪Y)SUP(X)   ×(1−SUP(X∪Y)|D|), α+β=1∧α,  β>0,

where *α*, *β* are two regulating coefficients with the interval [0,1].

From (20), we can see that the interestingness of a rule is the linear combination of the confidence and the complement of the support. As the two parts and two regulating coefficients all belong to the interval of [0,1], the interestingness of a rule lies also in the interval of [0, 1]. Meanwhile, it can also be seen that if the confidence keeps invariable, the support is the less, the interestingness is the larger, and vice versa; and when the support is fixed, the confidence is the larger, the interestingness is the larger, and vice versa. This is just in accordance with the definition of the interestingness of a rule.

From the above-mentioned, it can be seen that only by means of the attribute indices can all metrics to evaluate a rule be calculated out. Namely, the calculations of all metrics do not need to scan database any further, but only fetch from the created attribute indices. Therefore, there is no doubt that the proposed method can highly improve the performance of algorithm. 

### 3.2. Fitness Function

Evolutionary algorithm, EA, is a promising approach to find Pareto-optimal solutions. It uses a fitness function to guide the population members to converge toward the Pareto frontier. A well-known fitness function is the weighted sum of the objective function

(21) $$\begin{array}{c} \text{fitness}=\omega_{1}f_{1}\left(x\right)+\omega_{2}f_{2}\left(x\right)+\cdots +\omega_{M}f_{M}\left(x\right), \end{array}$$

where *ω*_1_, *ω*_2_,…, *ω*_*M*_ are nonnegative weights such that *ω*_1_ + *ω*_2_ + ⋯+*ω*_*M*_ = 1. We call *w* = (*ω*_1_, *ω*_2_,…, *ω*_*M*_) a weight vector.

If an EA uses one weight vector to compose one fitness function, there is only one search direction. To overcome this shortcoming, multiple weight vectors can be used to compose multiple fitness functions, so that there are multiple search directions. Leung and Wang applied the uniform design to compose multiple fitness functions, such that multiple search directions are scattered uniformly toward the Pareto frontier in the objective space. This method is as follows [23].

Firstly, normalize each objective function as follows:

(22) $$\begin{array}{c} h_{i}\left(x\right)=\frac{f_{i}\left(x\right)}{\text{ma}{\text{x}}_{y\in \psi }\left\{\left|f_{i}\left(y\right)\right|\right\}}, \end{array}$$

where *ψ* is a set of points in the current population and *h*_*i*_(*x*) is the normalized objective function.

Then compose *D* fitness functions for any given *D*, where the *i*th fitness function is given by (1 ≤ *i* ≤ *D*):

(23) $$\begin{array}{c} {\text{fitness}}_{i}=\omega_{i,1}h_{1}\left(x\right)+\omega_{i,2}h_{2}\left(x\right)+\cdots +\omega_{i,M}h_{M}\left(x\right). \end{array}$$

Let *w*_*i*_ = (*ω*_*i*,1_, *ω*_*i*,2_,…, *ω*_*i*,*M*_). The uniform design is applied to select the weight vectors *w*_1_, *w*_2_,…, *w*_*D*_ as follows. In the objective space, each objective function is treated as one factor and hence there are *M* factors. Assume *D* weight vectors and hence there are *D* levels. The uniform array *U*(*M*, *D*) is applied to determine *ω*_*i*,*j*_ for any and 1 ≤ *i* ≤ *D* and 1 ≤ *j* ≤ *M* as follows:

(24) $$\begin{array}{c} \omega_{i,j}^{}=\frac{U_{i,j}}{\sum_{j=1}^{M}U_{i,j}}. \end{array}$$

The equation can ensure the square sum of the weight for each fitness function to be one.

The *D* weight vectors *w*_1_, *w*_2_,…, *w*_*D*_ can provide *D* search directions. Using the uniform design to select the *D* weight vectors can ensure the *D* search directions to be scattered uniformly toward the Pareto frontier in the objective space.

In the proposed method, there are 4 objective functions, namely, *M* = 4, and (23) can be modified as follows:

(25) fitnessi=ωi,1confidence+ωi,2coverage +ωi,3comprehensiblity +ωi,4interestingness.

### 3.3. Encoding and Decoding

An association rule of the form *X* → *Y* can be represented as a chromosome, among which, each gene represents an attribute in the database. The itemset *X* and *Y* are, respectively, called the antecedent and consequent of a association rule. In general, a rule only contains a part of attributes, and the length, antecedent, and consequent, of the various rules are all variable. Therefore, it is a very urgent issue how to code a chromosome for the various rules.

 However, from the viewpoint of each attribute, the above problem can be easily handled. The existence of an attribute in an association rule can be classified into three situations as follows.

- The attribute does not exists in the rule;
- the attribute exists in the antecedent *X*;
- the attribute exists in the consequent *Y*.

It can be noted that there is not the situation that the attribute exists in both the antecedent and consequent, the reason of which is *X*∩*Y* = Φ in the definition of an association rule.

If the above situations are, respectively, coded as 0, 1, and 2, then the chromosome representing a rule can contain each attribute whose value is 0, 1, or 2. Therefore, the length of the chromosome is fixed and is equal to the number of attributes in the database.

The decoding of a chromosome is the reverse process of the coding. Namely, each gene, whose value is 0, 1 or 2, is translated as one of the above three situations. The index of the gene whose value is equal to 1 or 2, respectively, decoded as the antecedent and the consequent. The pseudocode of decoding is illustrated in Pseudocode 3.

An example of coding and decoding is described as follows.

**Example 4**

Assume the database with 6 attributes (*A*, *B*, *C*, *D*, *E*, *F*). An association rule *BC* → *E* can be coded as 011020. A chromosome 101022 can be decoded as the antecedent {1,3} and consequent of {5,6}, namely, the corresponding rule is *AC* → *EF*.

### 3.4. Initialization

Assume the size of the population and the number of attributes in the database to be *M*_*P*_ and *N*, an *M*_*P*_-by-*N* integer matrix is randomly generated. In the matrix, the value of each element is equal to 0, 1, or 2, which present three situations mentioned in Section 3.3. The matrix is the initialized population. The pseudocode of the initialization of a population is shown in Pseudocode 4. 

In Pseudocode 4, the function *isvalid* is to judge whether a chromosome representing a rule is valid or not. A valid rule is that the size of the antecedent and consequent of a rule are both larger than 0. Namely a chromosome is valid, if and only if the genes whose value is 1 and 2 in the chromosome are both larger than 0. For instance, the chromosomes such as 22000, 00000, and 01010 are all invalid, and those such as 11020 and 01012 are all valid. 

### 3.5. Crossover Operator of Variable Length

In order to ensure that each gene in chromosome has as many chances as possible to implement crossover operation, a crossover operator with variable lengths and positions is designed as follows. 

First generate a random integer *N*_1_, which represents the number to exchange genes, where *N*_1_ < *N*, *N* is the number of attributes in a database or the number of the genes in a chromosome. Then generate two random integer vectors of length *N*_1_, which represent the positions to exchange genes. Lastly, the genes of the corresponding positions in two chromosomes are exchanged with each other to generate two novel offspring. 

For example, two chromosomes *C*_1_ and *C*_2_ containing 10 genes are illustrated in Table 3(a). The steps of implementing crossover operator on two chromosomes *C*_1_ and *C*_2_ are as follows.

- Generate a random integer *N*_1_ = 5 which is less than 10.
- Two random integer vectors pos_1_ = {2,4, 7,8, 10} and pos_2_ = {1,3, 4,6, 8} are generated.
- Those genes whose positions are pos_1_ in *C*_1_, and those genes whose positions are pos_2_ in *C*_2_, are exchanged with each other.

The results of implementing crossover operation are illustrated in Table 3(b). Notice those genes located at the asterisk have been exchanged.

The pseudocode of the crossover function is shown in Pseudocode 5. Here, the function *isvalid* can be referred to Section 3.4. Only valid rule can be taken into the offspring.

### 3.6. Selection Scheme for Crossover Operation

The above crossover operation needs to firstly select some chromosomes from the population. A new selection scheme is designed as follows. For each pair chromosomes, one is selected randomly, another is chosen as the best one of chromosomes from various directions. These directions are provided by the weight vectors selected using the uniform design. The detailed steps are as follows.

First randomly select *K*_1_ chromosomes to a set *A* from the population according to the probability of crossover. Assume the number of the needed weight vectors is *D*_1_, where *D*_1_ is a prime number. If *K*_1_≥ *D*_1_, any *D*_1_ of the *K*_1_ chromosomes remain, the others are discarded. If *K*_1_<*D*_1_, randomly select *D*_1_ − *K*_1_ chromosomes to *A* in order that *A* contain *D*_1_ chromosomes. Next, apply the uniform design to select *D*_1_ − *M* weight vectors. The remainder *M* weight vectors are single objective weight vectors for *M* objective functions. Then, use (25) to generate *D*_1_ fitness functions, which can provide *D*_1_ search directions. Finally, adopt each fitness function to evaluate the quality of each chromosome in the population, and select the best one chromosome. Therefore, a total of *D*_1_ chromosomes are selected as another part of chromosomes to a set *B*. 

Each pair of *A* and *B* can be used to perform crossover operator. The pseudocode of the selection scheme for crossover operation is illustrated in Pseudocode 6. 

### 3.7. Mutation Operator

The function *mutate* is to handle the mutation operator. Its steps are as follows. Firstly fetch a chromosome according to the probability of mutation from a population and perform mutate operator on the chromosome in order to acquire a new one by calling the function mut. If the new chromosome is invalid, call the function mut again till the newly generated chromosome is valid. Next, the valid chromosome is taken as one of the offspring. Then, return to the first step and continue. Finally, return the generated offspring. The pseudocode of the mutation function is shown in Pseudocode 7. 

In Pseudocode 7, *mutate* function is used to handle mutation for the population contained many chromosomes by calling mut function. The mut function is used to handle mutation for the chromosome in the population. It firstly generates a random number. If the random number is less than the probability of mutation, the gene is changed into one of the remainder values in the set {0,1, 2}. The pseudocode of the mut function is shown in Pseudocode 8.

### 3.8. Elitist Selection or Elitism

Elitism means that elite individuals cannot be excluded from the mating pool of the population. A strategy presented can always include the best individual of the current population into the next generation in order to prevent the loss of good solutions that have been found. This strategy can be extended to copy the best *n* individuals to the next generation. This is explanation of the elitism. In evolutionary multiobjective optimization, elitism plays an important role [45]. Elitism can speed significantly up the performance of the genetic algorithm and help to achieve better convergence in multiobjective evolutionary algorithms, MOEAs [46]. MOEAs using elitist strategies tend to outperform their non-elitist counterparts [47]. Elitism usually has positive effects on both the convergence of solutions toward the Pareto front and the diversity along the Pareto front in MOEAs [48].

MOEAs often use two strategies to implement elitism. One maintains elitist solutions in the population, the other stores elitist solutions into an external secondary list and reintroduces them to the population. The former copies all non-dominated solutions in the current population to the next population, then fills the rest of the next population by selecting from the remaining dominated solutions in the current population. The latter uses an external secondary list to store the elitist solutions. The external list stores the non-dominated solutions found, and the list is updated in the next generation by means of removing elitist solutions dominated by a new solution or adding the new solution if it is not dominated by any existing elitist solution.

The work adopts the second strategy, namely storing elitist solutions to an external secondary list. Its advantage is that it can preserve and dynamically adjust all the non-dominated solutions set till the current generation. The pseudocodes of selecting elitist and updating elitist are, respectively, shown in Pseudocodes 9 and 10. 

### 3.9. Selection Scheme for Next Generation Combined with Elitism

After performing the crossover operator and mutation operator, we need to select some of the potential offspring to generate the new generation. Combining the elitism with the uniform design, the proposed algorithms design a new algorithm as follows.

We call the external secondary list the *elitist pool*. It stores the non-dominated solutions found till the current generation. Assume the size of the population and elitist pool, respectively, are *N*_*p*_ and *N*_pt_. If *N*_*p*_ ≤ *N*_pt_, then *N*_*p*_ nondominated solutions are randomly selected from the elitist pool as the next generation. Otherwise, all *N*_pt_ solutions in the elitist pool are taken as a part of the next generation, and the remainder *G* = *N*_*p*_ − *N*_pt_ chromosomes are selected as follows. 

Among the parents and the offspring generated by crossover and mutation, we select *G* of them to append to the next generation. In this selection, we adopt *D*_2_ fitness functions in order to realize *D*_2_ search directions, where *D*_2_ is a design parameter and it is prime. For each fitness function, each chromosome in the parents and offspring is evaluated using this fitness function and then the best ⌊*G*/*D*_2_⌋ or ⌈*G*/*D*_2_⌉ of them are selected, where ⌊*G*/*D*_2_⌋ mean the nearest integer less than or equal to *G*/*D*_2_, and ⌈*G*/*D*_2_⌉ mean the nearest integer larger than or equal to *G*/*D*_2_. Overall, a total of *G* chromosomes are selected to append to the next generation. Therefore, a total of *N*_*p*_ chromosomes are selected for the next generation.

### 3.10. The Steps of the Proposed Algorithm

The work proposes the attribute index and uniform design based multiobjective association rule mining with evolutionary algorithm, abbreviated as IUARMMEA. The steps of this algorithm are as follows.

**Step 1**

Firstly, load the whole database or a sample of records in the database *D* according to the capacity of the computer memory. Then, create the attribute index of each attribute in database by calling the function attrIdx described in Section 3.1. Finally, unload *D* to release the computer memory.

**Step 2**

Generate the initial population by calling the function “initialize” described in Section 3.4.

**Step 3**

Calculate several metrics values of the confidence, coverage, comprehensibility, and interestingness using (5), (7), (8), and (20), respectively. Choose all non-dominated solutions to the elitist pool from the initial populations by calling the function “paretocreate” described in Section 3.8.

**Step 4**

Select some chromosomes for performing the crossover operation from the population by calling the function “seleforcross” described in Section 3.6.

**Step 5**

Perform the crossover operation on the selected chromosomes by calling the function “crossover” described in Section 3.5.

**Step 6**

Perform the mutate operation on the selected chromosomes from the population in term of the probability of mutation by calling the function “mutate” described in Section 3.7.

**Step 7**

Regulate and update the non-dominated solutions in the elitist pool by calling the function “paretoupdate” described in Section 3.8. This step will compare the non-dominated solutions and the generated offspring after performing the crossover operator and mutation operator.

**Step 8**

Select some of the potential offspring to form the new generation by the selection scheme described in Section 3.9.

**Step 9**

Go to Step 4 and continue if the stop criterion is not met. Otherwise, go to Step 10.

**Step 10**

Decode all non-dominated solutions in the elitist pool to acquire the final association rules by calling the function “decode” described in Section 3.3.

## 4. Numerical Results

The proposed algorithm IUARMMEA is performed to test its performance and compare with the algorithm ARMMEA, which does not use the attribute index and uniform design.

### 4.1. Test Problems

We use six datasets to show the effectiveness and performance of IUARMMEA. The specifications of six datasets are described in Table 4. They represent various kinds of domains and include both dense and non-dense datasets, as well as various numbers of items. The first five datasets are from UCI repository [49]. The last dataset was generated using the generator from the IBM Almaden Quest research group. It can be acquired from the workshop on frequent itemset mining implementations [50]. 

For each dataset, the categorical attribute is converted or divided into boolean attribute in terms of each attribute and its various values. For instance, assume an attribute *x* can take any of the set {“*a*”,“*b*”,“*c*”,“*d*”,“*e*”} in a categorical dataset. Therefore, *x* can be divided into 5 attributes, such as *x*_1_, *x*_2_, *x*_3_, *x*_4_, *x*_5_. For each transaction, if *x* = *a*, then *x*_1_ = 1, otherwise *x*_1_ = 0; If *x* = *b*, then *x*_2_ = 1, otherwise *x*_2_ = 0, and so on. 

However, it can be noted that the gene in the chromosome only take one of the divided attributes, namely, only one attribute can be larger than 0 in the divided attributes. This is because the divided attributes are mutually exclusive. Therefore, the evolution, initialization, and evaluation of the population must consider the situation.

### 4.2. Parameter Values

The parameters of the proposed algorithm are as follows.

- *Population Size*: the population size is 100.
- *Parameters for Crossover and Mutation*: we adopt *p*_*c*_ = 0.9, *N*_1_ = 23 and *p*_*m*_ = 0.5.
- *Parameters for Interestingness*: the regulating coefficient *α* is 0.5.
- *Parameters for Selection*: *D*_1_ = 31.
- *Stopping Condition*: the algorithm terminates if the number of iterations is larger than the given maximal value 10.

### 4.3. Results

For each test problem, we perform 3 independent executions and calculate the average values of the following results, the number of scanning database, the number of comparing transactions, the number of comparing attribute indices, and execution times. Tables 5 and 6, respectively, show these average values of two algorithms.

Tables 5 and 6 indicate that IUARMMEA compared with ARMMEA, the number of scanning database is very little and can be disregarded. This is because in ARMMEA algorithm, each chromosome and each offspring generated by crossover and mutation need to scan database to calculate the support count of the antecedent, consequent, and the whole rule, while in IUARMMEA algorithm, all chromosomes and offsprings do not need to scan database any further, and only need to scan database once to create the attribute index. For the dataset T40I10D100, as the number of transactions and attributes is very large, it is loaded in three batches according to the capacity of the computer memory. Therefore, the number of scanning database is 3.

The number of comparing transactions is the product of the number of scanning database and the number of transactions in database, since scanning database once is to compare each transaction in database with each part of the rule. As the algorithm ARMMEA has not the attribute index, the number of comparing indices is certainly 0. From Table 5, it can be seen that there is a relationship between the number of comparing indices and the number of the undivided attributes. This is because several metrics of a rule need to compare the attribute indices of the undivided attributes.

Tables 5 and 6 also indicate that the execution times of IUARMMEA are significantly less than those of ARMMEA. The former really outperforms the latter. In the meanwhile, it can be seen that the execution times have relation to not only the number of comparing the indices but also the lengths of attribute index. For example, the dataset mushroom compared with chess, the number of comparing indices is less, but the execution times are even longer. This is because the length of attribute index is much larger.

**Figure:**
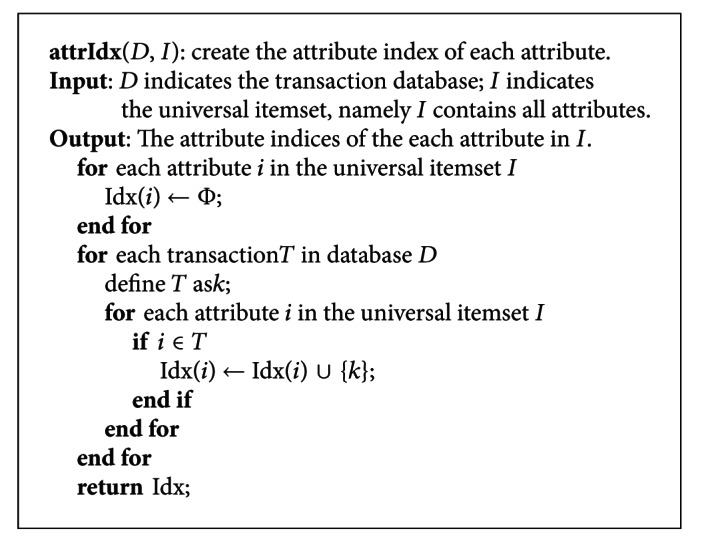
Pseudocode of creating the attribute index.

**Figure:**
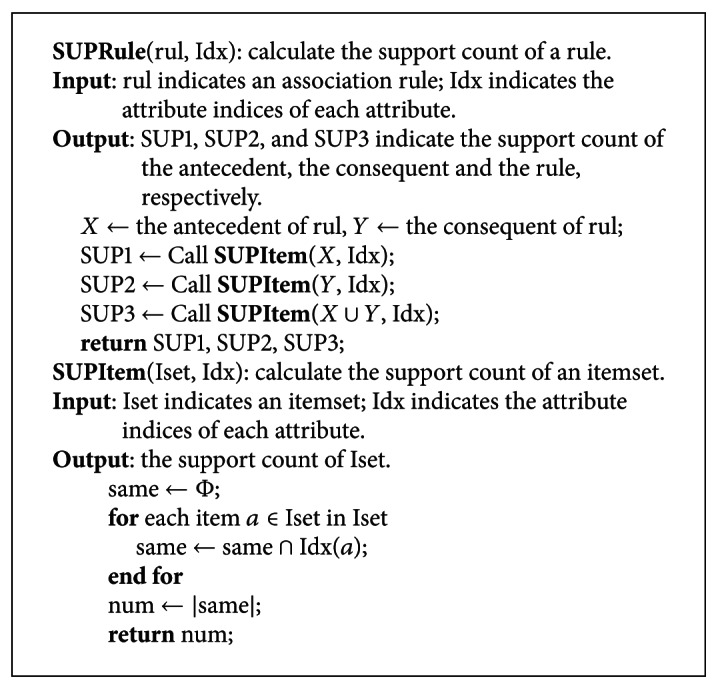
Pseudocode of calculating the support count.

**Figure:**
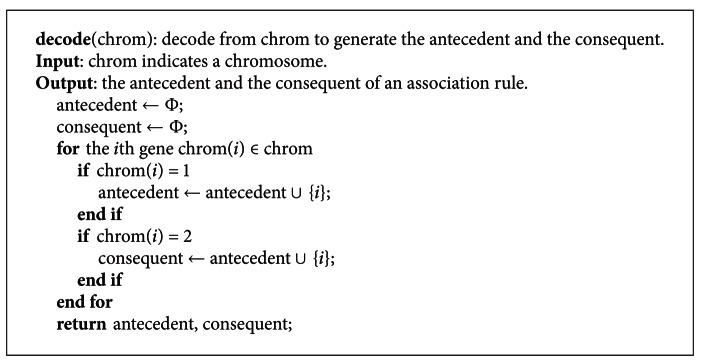
Pseudocode of decoding.

**Figure:**
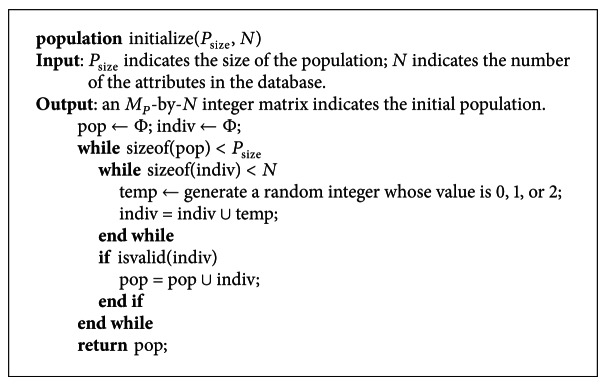
Pseudocode of the initialization.

**Figure:**
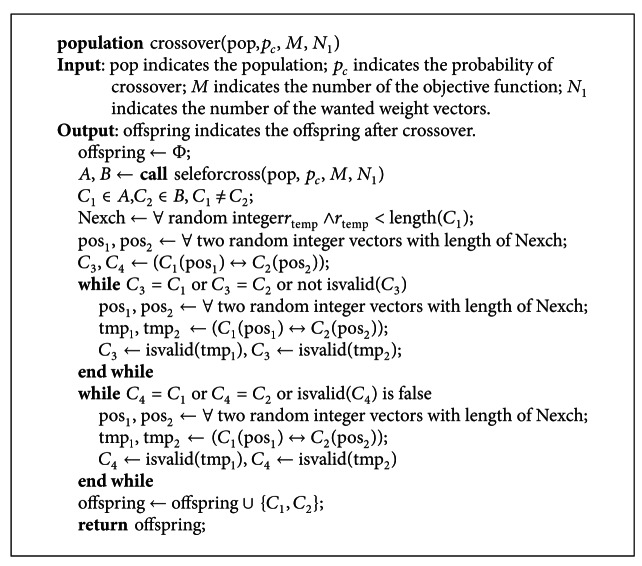
Pseudocode of the crossover operator.

**Figure:**
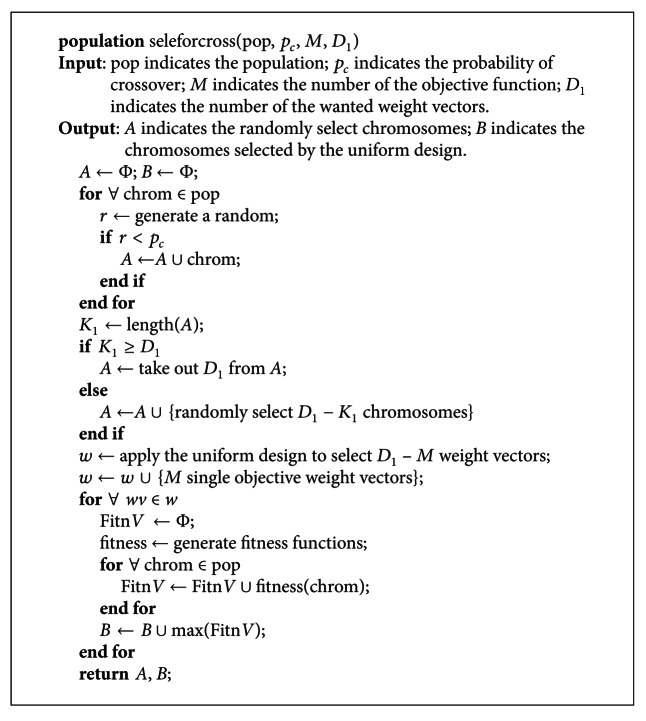
Pseudocode of the selection scheme for crossover operation.

**Figure:**
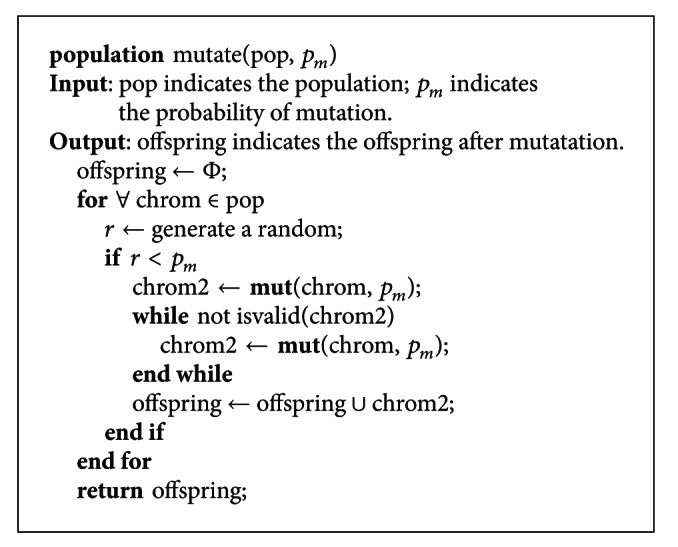
Pseudocode of the mutate operator.

**Figure:**
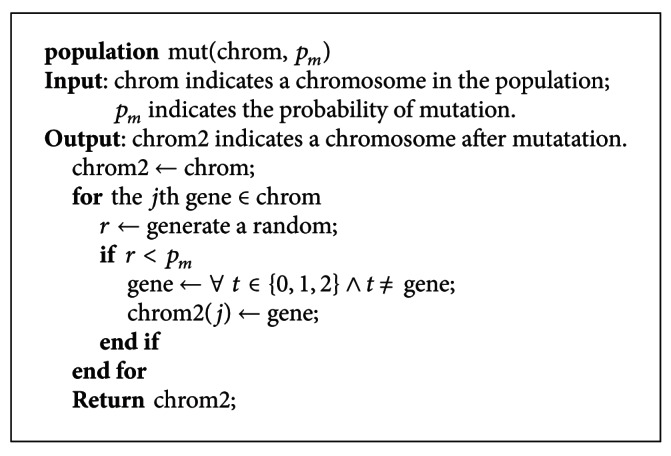
Pseudocode of the mut function.

**Figure:**
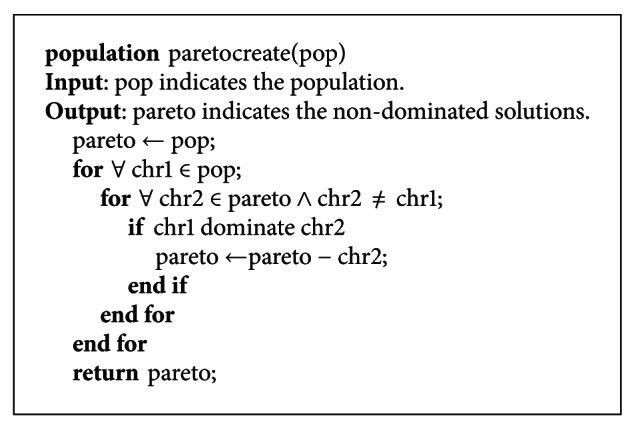
Pseudocode of selecting elitist.

**Figure:**
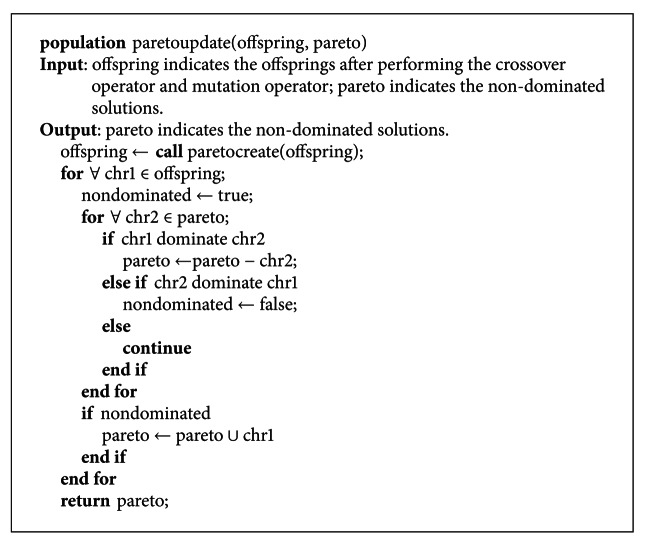
Pseudocode of updatting elitist.

## 5. Conclusion and Future Work

In this paper, we present a method of multiobjective association rule mining based on the attribute index and uniform design. The proposed method only scans database once to create the attribute index and uses it to replace repeatedly scanning database. This significantly reduces the number of comparisons and time consumption, and improves the performance of the algorithms.

This algorithm is going on for further enhancement and improvement. Attempt is to extend it to immediately use the categorical or numeric dataset rather than converting them into Boolean dataset.

## Figures and Tables

**Table 1:** Values of the parameter σ for different number of factors and different number of levels per factor.

Number of levels per factors	Number of factors	*σ*
5	2~4	2
7	2~6	3
11	2~10	7
	2	5
13	3	4
	4~12	6
17	2~16	10
19	2~3	8
	4~18	14
	2, 13~14, 20~22	7
23	8~12	15
	3~7, 15~19	17
	2	12
	3	9
29	4~7	16
	8~12, 16~24	8
	13~15	14
	25~28	18
31	2, 5~12, 20~30	12
	3~4, 13~19	22

**Table 2:** An example of transation database.

Transaction	Containing items
*T*_1_	*i*_1_, *i*_3_, *i*_5_
*T*_2_	*i*_4_, *i*_5_
*T*_3_	*i*_1_, *i*_2_, *i*_4_, *i*_5_
*T*_4_	*i*_2_, *i*_3_, *i*_4_, *i*_5_
*T*_5_	*i*_1_, *i*_3_, *i*_5_, *i*_6_
*T*_6_	*i*_1_, *i*_3_, *i*_4_, *i*_5_, *i*_6_

**Table:** (a) Before crossover

*C*_1_	*A*_1_	*A*_2_	*A*_3_	*A*_4_	*A*_5_	*A*_6_	*A*_7_	*A*_8_	*A*_9_	*A*_10_
pos_1_		2		4			7	8		10

*C*_2_	*B*_1_	*B*_2_	*B*_3_	*B*_4_	*B*_5_	*B*_6_	*B*_7_	*B*_8_	*B*_9_	*B*_10_

pos_2_	1		3	4		6		8		

**Table:** (b) After crossover

*C*_1_	*A*_1_	*B*_1_	*A*_3_	*B*_3_	*A*_5_	*A*_6_	*B*_4_	*B*_6_	*A*_9_	*B*_8_
pos_1_		∗		∗			∗	∗		∗

*C*_2_	*A*_2_	*B*_2_	*A*_4_	*A*_7_	*B*_5_	*A*_8_	*B*_7_	*A*_10_	*B*_9_	*B*_10_

pos_2_	∗		∗	∗		∗		∗		

**Table 4:** The specifications of data sets.

Dataset	Number transactions	Number attributes
Balance scale	625	23
Solar flare	1066	48
Chess	3196	75
Mushroom	8124	119
Nursery	12960	32
T40I10D100K	100000	942

**Table 5:** The averages of several results for IUARMMEA.

Dataset	Number scanning database	Number comparing transactions	Number comparing indices	Execution times (sec)
Balance scale	1	625	8406	52.04
Solar Flare	1	1066	17047	99.36
Chess	1	3196	43683	282.81
Mushroom	1	8124	27123	363.05
Nursery	1	12960	9121	296.87
T40I10D100K	3	300000	85447	712.62

**Table 6:** The averages of several results for ARMMEA.

Dataset	Number scanning database	Number comparing transactions	Number comparing indices	Execution times (sec)
Balance scale	2380	1487500	0	121.39
Solar flare	2326	2479516	0	232.66
Chess	2320	7414720	0	626.78
Mushroom	2320	18847680	0	1183.06
Nursery	2378	30818880	0	1312.82
T40I10D100K	6885	229500000	0	1937.53
